# Stem Cell Therapy for Myocardial Infarction and Heart Failure: A Comprehensive Systematic Review and Critical Analysis

**DOI:** 10.7759/cureus.59474

**Published:** 2024-05-01

**Authors:** Mohamed R Abouzid, Ahmed Muaaz Umer, Suman Kumar Jha, Usman A Akbar, Own Khraisat, Amr Saleh, Kareem Mohamed, Sadaf Esteghamati, Ibrahim Kamel

**Affiliations:** 1 Internal Medicine, Baptist Hospitals of Southeast Texas, Beaumont, USA; 2 Internal Medicine Residency, Camden Clark Medical Center, Parkersburg, USA; 3 Internal Medicine, Sheer Memorial Adventist Hospital, Banepa, NPL; 4 Internal Medicine, Camden Clark Medical Center, Parkersburg, USA; 5 Internal Medicine, King Hussein Medical City, Amman, JOR; 6 Cardiovascular Medicine, Yale School of Medicine, New Haven, USA; 7 Internal Medicine, University of Missouri Kansas City, Kansas City, USA; 8 Internal Medicine, University of La Verne, La Verne, USA; 9 Internal Medicine, Steward Carney Hospital, Boston, USA

**Keywords:** adipose tissue-derived mscs, angiogenesis, cardiac repair, cardiac stem cells, challenges, induced pluripotent stem cells, ischemic heart disease, mesenchymal stem cells, stem cells, umbilical cord-derived mscs

## Abstract

In exploring therapeutic options for ischemic heart disease (IHD) and heart failure, cell-based cardiac repair has gained prominence. This systematic review delves into the current state of knowledge surrounding cell-based therapies for cardiac repair. Employing a comprehensive search across relevant databases, the study identifies 35 included studies with diverse cell types and methodologies. Encouragingly, these findings reveal the promise of cell-based therapies in cardiac repair, demonstrating significant enhancements in left ventricular ejection fraction (LVEF) across the studies. Mechanisms of action involve growth factors that stimulate angiogenesis, differentiation, and the survival of transplanted cells. Despite these positive outcomes, challenges persist, including low engraftment rates, limitations in cell differentiation, and variations in clinical reproducibility. The optimal dosage and frequency of cell administration remain subjects of debate, with potential benefits from repeated dosing. Additionally, the choice between autologous and allogeneic stem cell transplantation poses a critical decision. This systematic review underscores the potential of cell-based therapies for cardiac repair, bearing implications for innovative treatments in heart diseases. However, further research is imperative to optimize cell type selection, delivery techniques, and long-term efficacy, fostering a more comprehensive understanding of cell-based cardiac repair.

## Introduction and background

Ischemic heart disease (IHD) is a significant worldwide health problem, resulting in 17.9 million fatalities per year [1,2]. Despite recent improvements in treatment, a significant number of individuals still experience persistent ischemia and congestive heart failure (CHF). Despite the recent advances in the management of IHD in the form of medications or coronary revascularization, many patients are left with chronic ischemia and nearly one-third of them end up developing congestive heart failure (CHF) [3]. The available medical treatment for CHF can only address its symptoms without correcting the underlying pathology.

Stem cell therapy, an innovative medical approach, has demonstrated encouraging outcomes in the regeneration of damaged heart muscle tissue and the restoration of its ability to contract. Cardiac stem cells (CSCs), mesenchymal stem cells (MSCs), and bone marrow-derived stem cells have demonstrated encouraging outcomes [4]. The suggested interventions for this therapy encompass anti-fibrotic properties, immunomodulatory effects, neovascularization, promotion of endogenous heart regeneration, and mitigation of unfavorable ventricular remodeling [5]. The primary emphasis of research investigations in the domain of cell-based therapy revolves around determining the most suitable cell type for delivery, the safest method of administration, the ideal cell dosage, the clinical feasibility, and addressing problems such as cell homing or survival [6].

The main aim of this systematic review is to shed light on the current status of stem cell therapy as a potential breakthrough in cardiac repair and the treatment of heart failure. We discuss the biology of several stem cell types, mechanisms of cardiac regeneration, translational findings in clinical settings, challenges with possible solutions, and different data from preclinical and clinical studies.

## Review

Methods

Search Strategy and Study Selection

The Preferred Reporting Items for Systematic Reviews and Meta-Analyses (PRISMA) standards were adhered to during the process of preparing this systematic review. We conducted a systematic review of all published studies from January 2007 till December 2022 investigating stem cell therapy for heart failure and myocardial infarction (MI). The search for studies was conducted exclusively in the English language, utilizing databases such as Medline, PubMed, and Scopus. Additionally, gray literature sources such as the Centers for Disease Control and Prevention (CDC) and the World Health Organization (WHO) were investigated.

The process of conducting a literature search and screening approach, selecting studies, extracting data, and assessing the risk of bias was carried out according to established inclusion criteria. These procedures were overseen by the senior author to ensure the maintenance of quality. The search strategy included keywords and Medical Subject Headings (MeSH) terms related to ischemic heart disease, heart failure, cell therapy, stem cells, and cardiac repair. The primary endpoint is left ventricular ejection fraction (LVEF), and the secondary endpoints include infarct size, ventricular wall thickness, end-systolic volume, and end-diastolic volume. The database search yielded several types of scholarly literature, including randomized control trials, observational studies, review articles, meta-analyses, and original publications, which focused on both animal models and human subjects.

All studies were chosen based on their compliance with the inclusion criteria. Two independent authors then assessed the content of the studies to determine their eligibility. Further discrepancies were addressed through a collective deliberation, during which a choice was made to either incorporate or exclude a study based on pre-established criteria. The reviewer conducted a quality assessment to ascertain the presence of a well-defined research topic with precise outcomes, a clear delineation of inclusion and exclusion criteria, robust methodology, potential for generalizability, and acknowledgment of limitations.

*Inclusio*​​​​*n and Exclusion Criteria*

For the inclusion criteria, we will consider studies published from January 2007 to December 2022, in English, including randomized controlled trials, animal studies, systematic reviews, and meta-analyses.

Exclusion criteria encompass studies published more than 16 years ago, studies in languages other than English, case studies, editorials, and studies not addressing LVEF as an endpoint.

Data Extraction

Two independent reviewers conducted a rigorous screening and data extraction process. Any discrepancies were resolved through discussion and consensus. Extracted data included study author, sample size, type of stem cells, mode of delivery, follow-up period, and outcomes. 

Statistical Analysis

Due to the substantial heterogeneity among the included studies, a meta-analysis was not feasible. Therefore, a narrative synthesis of the findings focused on the main outcomes and trends observed across the selected studies.

Results

The study selection process was systematically conducted in adherence to PRISMA guidelines. The search initially yielded a total of 1457 records, which were deduplicated, resulting in 721 duplicate records being removed. Subsequently, the remaining 736 unique records underwent screening for relevance based on inclusion and exclusion criteria, leading to the exclusion of 652 records that did not meet these criteria. The remaining 84 records were further evaluated for eligibility, and 49 were excluded based on predefined criteria.

**Figure 1:**
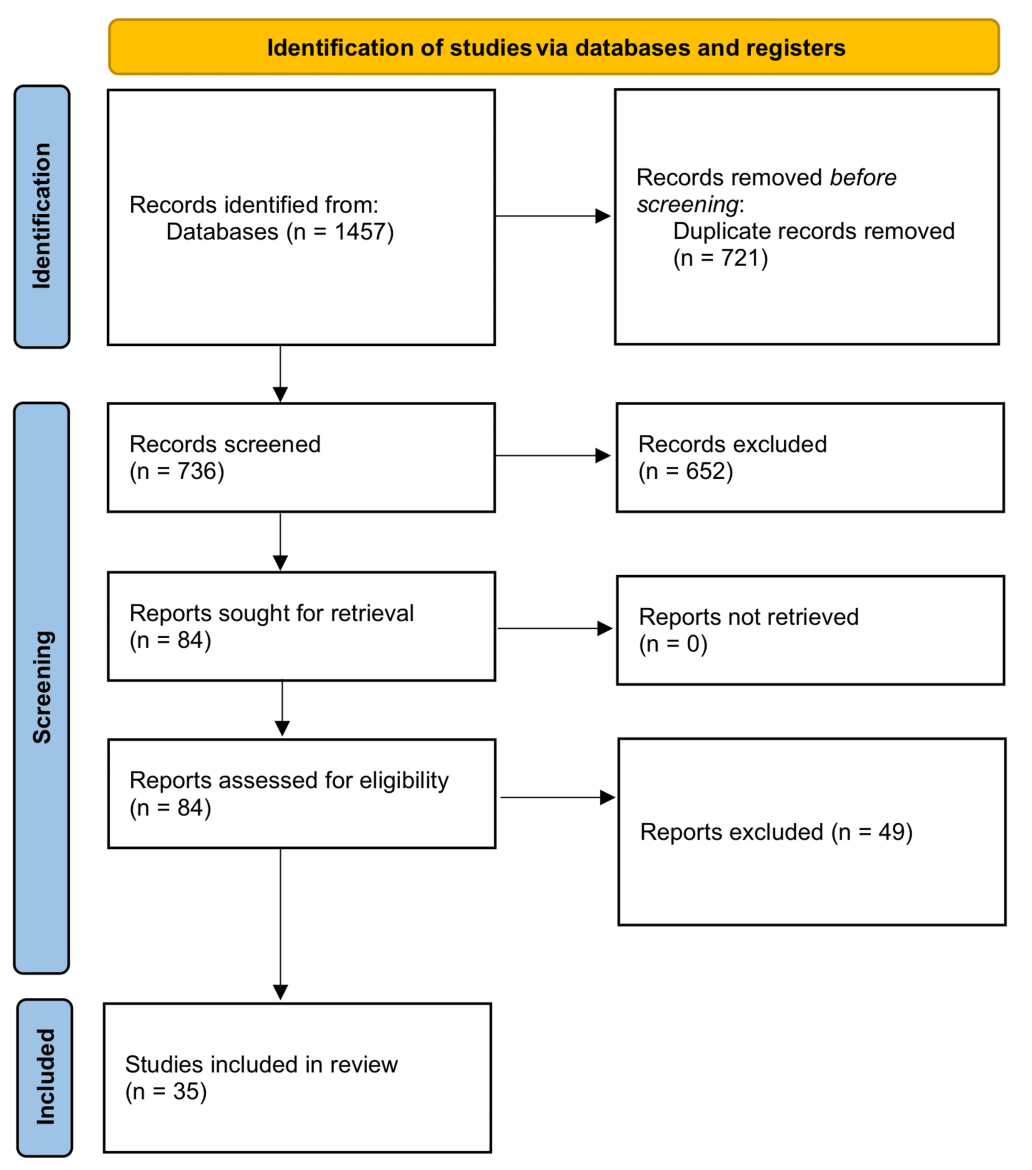
PRISMA flow diagram PRISMA: Preferred Reporting Items for Systematic Reviews and Meta-Analyses.

Ultimately, 35 studies met the inclusion criteria and were included in this systematic review [7-41]. A PRISMA flow diagram (Figure 1) was used to visually represent the selection process, illustrating the number of records at each stage, from the initial search to the inclusion of studies. This approach ensured a transparent and systematic approach to study selection in line with PRISMA standards. Cell-based therapies appear to have promise for cardiac repair, with considerable improvements in LVEF found across investigations. The increase in LVEF indicates improved heart function following cell-based treatments. The characteristics and summary of the included studies are illustrated in Table 1. 

**Table 1:** Summary of the included studies. CSC: cardiac stem cell; MSC: mesenchymal stem cells; BM: bone marrow; LVEF: left ventricular ejection fraction; VEGF: vascular endothelial growth factor.

Study	No. of patients	Type of stem cells	Dose	Mode of delivery	Follow-up period	Outcome
Butler et al., 2017 [7]	Total = 22; Treated group = 10, Control group= 12	Ischemia-tolerant human donor allogeneic MSCs (itMSCs)	1.5 × 10^6^ cells/kg	Intravenous	90 days	-itMSC therapy was safe - Improvement in the health status and functional capacity - No significant differences in mortality, hospitalization, or serious adverse event
Ulus et al., 2020 [8]	Total = 42; Treated group = 26; Control group = 16	Human umbilical cord-derived mesenchymal stromal cells or bone marrow-derived MSCs	21∼26 × 10^6^	Intramyocardial	12 months	There was a decline in NT-proBNP levels in the treated group. The HUC-MSC group specifically showed an increase in LVEF. Additionally, decreases in necrotic myocardium were observed.
Bolli et al., 2011 [9]	(Stage A) Treated group = 9; Control group = 4. (Stage B) Treated group = 7; Control group = 3	Autologous CSCs	Based on the number and location of the infarcts (not to exceed a total of 1 million cells)	Intracoronary	Four-month	- Safe - Increase in LVEF - Decrease in infarct size
Chin et al., 2011 [10]	Group A: Five had ischemic dilated cardiomyopathy (DCM) unlikely to benefit from revascularization alone. Group B: Two patients had previous revascularization and three had non-ischemic DCM	Mesenchymal stromal cells	Group A received 0.5-1.0 × 10^6^ MSC/kg body weight - Group B received 2.0-3.0 × 10^6^ MSC/kg body weight	Group A: Intramyocardial Group B: Intracoronary	Six-week, three-month, six-month, and 12-month	- Safe and feasible - Increase in LVEF - Decrease in infarct size
Chugh et al., 2012 [11]	Treated group = 20; Control group = 13	c-kit+ CSCs	Eighteen treated patients received 1 × 10^6^ cells and two received 5 × 10^5^ cells	Intracoronary	Four- and 12-month	- Improvement in both global and regional LV function. - Reduction in infarct size. - Persistent increase in viable tissue
Gao et al., 2015 [12]	Total patients = 116; Treated group = 58; Control group = 58	Wharton’s jelly-derived mesenchymal stem cells (WJ-MSCs)	6 × 10^6^ WJ-MSCs	Intracoronary	18-month	- Increase in the LVEF - Increase in the myocardial viability and perfusion within the infarcted region
Guijarro et al., 2016 [13]	Ten patients	MSCs	61.5 × 10^6^ cells per patient	Intramyocardial	One-month, 12-month, and two-year	- Safe - Improvements in cardiac performance, LV remodeling, and patient functional status
Hare et al., 2017 [14]	Thirty-seven patients in a 1:1 ratio	MSCs	100 million MSCs	Transendocardial	Baseline, 30 days, and three-, six-, and 12-month	- Allogeneic stem cell injection is safer and more effective than autologous stem cells for nonischemic DCM - Pivotal trials of allogenic MSCs are required according to these results
Bartunek et al., 2017 [15]	Forty-eight patients	Autologous bone marrow-derived and cardiopoietic MSCs	733 x 10^6^ cells	Endomyocardial	- The primary endpoint (feasibility/safety) at two-year follow-up. Secondary endpoints (cardiac structure/function and clinical performance) six months post-therapy	- Improved LVEF - Decrease in LV end-systolic volume - Improvement in exercise tolerance, quality of life, and physical performance
Hare et al., 2009 [16]	Fifty-three patients	BM-MSCs	0.5, 1.6, and five million cells/kg	Intravenous	Six-month	- Safe and feasible - Increase in LVEF - Reversal of ventricular remodeling - Improved overall performance
CONCERT-HF Trial., 2021 [17]	Total patients = 18; Treated group = 9; Control group = 9	MSCs + CSCs	MSCs dose = 150×10^6^ cells CSCs dose = 5×10^6^ cells	Intramyocardial	Baseline, one-month, six-month, and 12-month	N/A
Li et al., 2015 [18]	Fifteen patients	UC-MSCs	Low-dose 3 x 10^6^, mid-dose 4 x 10^6^ and high-dose 5 x 10^6^ groups	Intracoronary	12-month and 24-month	- Safe and feasible - Increase in LVEF and decrease in infarct size
TAC-HFT randomized trial, 2014 [19]	Total patients = 65 MSC; Treated group = 19; Control group = 11 BMC: Treated group = 19; Control group = 10	MSCs and bone marrow mononuclear cells (BMCs)	N/A	Transendocardial	One-month and 12-month	- Both are generally safe without serious adverse events following injection - Improved functional status with MSCs - Decrease in infarct size with MSCs - Increase in LVEF
CADUCES Trial, 2014 [20]	Treated group = 17 (plus 1 infused off-protocol 14 months post-MI), Control group = 8	Cardiosphere-derived cells (CDCs)	12.5 to 25 × 10^6^	Intracoronary	One-year	- Decrease in scar area - Increase in viable tissue - Improved LVEF and cardiac function - Safe
Houtgraaf et al., 2012 [21]	Total = 14; Treated group = 10, Control group = 4	Adipose tissue-derived regenerative cells (ADRCs)	20 million ADRCs	Intracoronary	Six-month	- Improvement in LVEF and cardiac function - Decrease in infarct area - Improvement in cardiac perfusion - Safe and feasible without serious adverse effects
PRECISE Trial, 2014 [22]	Total = 27; Treated group = 21, Control group = 6	Adipose-derived regenerative cells (ADRCs)	0.4 x 10^6^ ADRCs/kg, 0.8 x 10^6^ ADRCs/kg, and 1.2 x 10^6^ ADRCs/kg (escalating doses)	Transendocardial	One-, three-, six-, 12-,18- up to 36-month	- Safe and feasible - Improvement in total left ventricular mass and cardiac function - Improvement in cardiac perfusion - Improvement in exercise capacity
MyStromalCell Trial, 2012 [23]	Sixty patients	AD-MSCs	N/A	Intramyocardial	After four, 12, and 26 weeks and then after one, two, and three years	- Decrease in infarct size in the LV size - Improvement in maximum oxygen consumption and exercise tolerance - Improvement in myocardial perfusion - Increase in LVEF - Improved symptoms
Ascheim et al., 2014 [24]	Thirty patients were randomized in a ratio of 2:1	Allogeneic mesenchymal precursor cells (MPCs)	25 million MPCs	Intramyocardial	Twelve-month or until transplant, whichever came first	- Safe with no difference in serious adverse events - There is a potential signal of efficacy that needs further evaluation
Musialek et al., 2015 [25]	Ten patients	WJ-MSCs	30 × 10^6^ WJ-MSCs	Intracoronary	12-month	- Safe and feasible without major adverse effects
RIMECARD Trial, 2017 [26]	Treated group = 15; Control group = 15	UC-MSCs	1 × 10^6^ cells/kg	Intravenous	Three-, six-, and 12-month	- Increase in LVEF - Improvements of New York Heart Association functional class - Improvement in quality of life
Zhao et al., 2015 [27]	Total patients = 59; Treated group = 30, Control group = 29	UC-MSCs	N/A	Intracoronary	Baseline, six-month, and 12-month	- Increase in six-minute walking distance of the treatment group - improvement of cardiac remodeling and cardiac function - Decrease in mortality rate
Sürder et al., 2016 [28]	Total patients = 200; Treated group= 133, Control group = 67	Bone marrow-derived mononuclear cells (BM-MNC)	N/A	Intracoronary	Baseline, and 12-month	- Overall, one-year mortality was low - Among patients with AMI and LV dysfunction, treatment with BM-MNC either five to seven days or three to four weeks after AMI did not improve LV function at 12 months, compared with control
SWISS-AMI, 2010 [29]	Total patients = 192; Treated group = 128, Control group = 64	BM-MNCs	Fifty milliliters of autologous bone marrow	Intracoronary	Baseline, four-month, 12-month, and 24-month	- Improve remodeling of the left ventricle after acute MI
TIME Randomized Trial, 2012 [30]	Total patients = 120; Treated group = 79, Control group = 41	BM-MNCs	150 × 10^6 ^BMCs or placebo	Intracoronary	Baseline, and six-month	- No complications were associated with intracoronary infusion. - No significant increase in LVEF
TOPCARE-AMI, 2011 [31]	Total patients = 55	CPC or BMC	N/A	Intracoronary	Five-year follow-up	Reduction in functional infarct size -Increment in LV-end diastolic volume
Wöhrle et al., 2010 [32]	Total patients = 42; BMC group = 29, Placebo group = 13	Autologous bone marrow cells (BMCs)	381 x 10^6^ mononuclear BMCs	Intracoronary	Baseline, one-month, and six-month	No positive evidence of intracoronary BMC versus placebo therapy with respect to LV ejection fraction, LV volume indexes, or infarct size
STAR-heart study, 2010 [33]	Total patients = 391; Treated group = 191, Control group = 200	BMC (Progenitor or stem cells)	80–120 mL of BMC	Intracoronary	Baseline, three-month, 12-month, and 60-month	-Significant improvement in hemodynamics (e.g., LVEF, cardiac index, exercise capacity, oxygen consumption, and LV contractility - Decrease in long-term mortality in the BMC-treated patients compared with the control group)
Stamm et al., 2007 [34]	Total patients = 55; Treated group = 35, Control group = 20	CD133+ bone marrow cell	2.95 × 10^7^ CD34^+^ cells	Intramyocardial	Baseline, and 12-month	- Intramyocardial delivery of purified bone marrow stem cells together with CABG surgery is safe. - Improvement in LVEF
REGENT Trial, 2009 [35]	Total patients = 200; Treated group= 160, Control group = 40	Bone marrow-derived unselected mononuclear cells (UNSEL) and selected CD34(+)CXCR4(+) cells (SEL)	N/A	Intracoronary	Baseline, and six-month	- No significant improvement in LVEF -Use of selected CD34^+^CXCR4^+^ cells in patients with significantly reduced LV function is safe, feasible, and needs further investigation
RENEW Trial, 2016 [36]	Total patients = 112; Treated group = 57, Control group = 55	Autologous CD34(+) cells	(10 × 0.2 ml) of 1 × 10^5^ auto-CD34^+^ cell/kg up to 1 × 10^7^ cells	Intramyocardial	Baseline, three-month, six-month, and 12-month	- Autologous CD34(+) cell therapy is safe - Due to early termination, RENEW was an incomplete experiment
IMPACT-CABG Trial, 2016 [37]	Total patients = 40; Treated group = 20, Control group = 20	Autologous CD133^+^ stem cells	0.5 × 10^6^-10 × 10^6^	Intramyocardial	Baseline, and six-month	- Safe and feasible - No significant improvement in LVEF
Qayyum et al., 2017 [38]	Total patients = 161; Treated group = 78, Control group = 83	Autologous VEGF-A_165_-stimulated adipose-derived stromal cells (ASCs)	10–15 injections of 0.2 mL of ASCs	Intracoronary	Baseline, three-month, and six-month	- Safe and feasible - Increase in exercise capacity in the ASCs group but not in the placebo group -Improvement in LVEF
POSEIDON Randomized Trial, 2012 [39]	Total patients = 31; Treated group = 31	MSCs	20 million, 100 million, or 200 million cells (five patients in each cell type per dose level)	Transendocardial	Baseline, six-month, 12-month, and 13-month	- Improvement in the patient functional capacity, quality of life, and LV remodeling - Low-dose concentration MSCs (20 million cells) produced the greatest reductions in LV volumes and increased LVEF
C-CURE Trial, 2013 [40]	Total patients = 46; Treated group = 32, Control group = 15	MSCs	N/A	Intramyocardial	Two-year follow-up	-Cardiopoietic stem cell therapy was feasible and safe with signs of improvement in chronic heart failure - Improved six-minute walk distance
Lee et al., 2014 [41]	Total patients = 80	MSCs	7.2±0.90 × 10^7^ cells	Intracoronary	Baseline and six-month	-Improved LVEF - Tolerable and safe - Modest improvement in LVEF at six-month follow-up

Discussion

Over the past few decades, animal and clinical studies have explored several types of stem cells to find the best one for cardiac repair and contractile function restoration [20,21]. Stem cell types employed in cardiac cell-based therapy are briefly discussed below.

MSCs

MSCs are stem cells that can self-renew, clone, and differentiate into mesenchymal and non-mesenchymal lineages. They can differentiate into cardiomyocytes, blood vessel endothelial cells, and smooth muscles. MSCs can be obtained from bone marrow (BM), peripheral blood, adipose tissue, umbilical cord, or synovial tissue. BM-MSCs have several benefits, but their use in animal experiments is controversial due to potential risks. MSCs exhibit surface markers but lack MHC class II and other costimulatory markers [42]. 

Umbilical cord-derived mesenchymal stem cells (UC-MSCs) can be collected from discarded placenta and release angiogenic, anti-apoptotic, and growth factors. They are chemoattractive and produce pro-angiogenic molecules, such as VEGF, hepatocyte growth factor (HGF), and angiopoietin. AD-MSCs, another type of mesenchymal stem cell, are an ideal autologous source of therapeutic regenerative cells. They are multipotent, pro-angiogenic, and can differentiate into cardiomyocytes and endothelial cells without immunologic issues when employed for cell-based therapy after myocardial infarction. AD-MSCs are easy to isolate in high quantities by liposuction and are widely available [43].

CSCs

Endogenous CSCs are an interesting discovery in heart regeneration medicine. This disproves the idea that the heart is terminally differentiated and that all cardiomyocytes cannot re-enter the cell cycle. However, the modest number of resident CSCs limits their therapeutic use. In addition, their endogenous regeneration potential cannot compensate for significant segmental myocardial loss in MI [44]. A mouse model using "mosaic analysis with double markers" showed that post-natal cardiomyocyte synthesis is rare and limited 50. CSCs include c-kit+, Sca-1+, Islet-1+, SP, Cs, and CDCs 11. Indeed, c-kit+ CSCs were the first heart CSC subtype identified, and they have shown promising effects after transplantation, reduction of ventricular remodeling, and cardiac function enhancement in animal investigations [45]. The SCIPIO trial, the first human phase 1, randomized controlled trial of autologous c-kit+ CSCs in heart failure patients, found that patients with ischemic heart failure who undergo coronary artery bypass grafting (CABG) can be treated with CSCs, which can be isolated from the heart during surgery [9]. 

In comparison to MSCs, CSCs differentiated into cardiomyocytes, had stronger angiogenic potential, improved engraftment, and balanced paracrine factor secretion. These superior qualities promote cardiac functioning more than extracardiac stem cells. Animal studies suggest dual stem cell transplantation is a promising cell-based therapy. CardioChimeras (CCs) generated by fusing CSCs and MSCs showed better cardiac healing and function than the injection of each cell type separately in an animal study by Pearl Quijada et al [46].

Other Low-Effective Stem Cell Types

Induced pluripotent stem cells (iPSCs) and human embryonic stem cells (hESCs) provide an unlimited source of tissue-specific cell types, such as cardiomyocytes. Despite ethical and social issues surrounding ESCs, iPSCs can produce atrial, nodal, and ventricular cardiomyocytes. Genetic reprogramming from adult somatic cells creates pluripotent iPSCs, which can self-renew and differentiate into many cell types, including cardiomyocytes. However, iPSCs are vulnerable in disease modeling and personalized medical treatment due to their ability to maintain patient-specific genomic, transcriptomic, proteomic, and metabolomic information. Immature hiPSCs are the main challenge, and novel treatments such as transient reprogramming factors may overcome this barrier. Human amniotic fluid (hAF) cells can multiply and develop into all embryonic germ layers without becoming teratomas. A two-week human study found that cardiopoietic AF cells upregulated cardiac transcription factors and expressed multipotency markers [47].

Routes of Stem Cell Delivery

Cell delivery is a crucial aspect of determining the safest and most effective way to deliver stem cells inside damaged cardiac muscle. There are several routes of cell delivery, including intracoronary, transendocardial, intravenous, and intramyocardial. 

The intracoronary route is an efficient method due to its safety, less invasiveness, and homogenous uniform distribution of transplanted stem cells into the targeted region of the heart. However, it has drawbacks such as the induction of further ischemia, the limitation of cell delivery in poorly perfused regions, and limited doses of stem cells [32,33,48].

The transendocardial route involves catheter-based injection of stem cells through the endocardium, which relies on electromechanical mapping (EMM) for precise identification and differentiation of infarcted and healthy myocardial tissue. It is a safe, highly effective, and less invasive method, but its efficacy is debatable in clinical trials [22,39].

The transcoronary venous route is another potential method for stem cell delivery to the infarcted heart, but it has limitations such as variability of coronary venous circulation among patients and tortuosity of coronary veins leading to difficulty passing through them [49]. The intravenous route seems to be the safest, easiest, and least invasive, but its efficacy decreases due to the lack of proper homing and implantation into the targeted area of the heart.

The pulmonary first-pass in the lungs is another concern that needs to be investigated [26]. A swine study suggested that intravenous injection of MSCs during the early post-infarct stage enhances regional myocardial perfusion and improves LVEF. However, clinical translation yielded conflicting results [50]. Intravenous injection of MSCs is generally safe and well-tolerated with some anti-inflammatory and immunomodulatory effects, but there was no significant difference in EF between treatment and placebo groups [50].

The surgical intramyocardial route, performed through thoracotomy during open-heart surgeries or as a separate procedure, offers the most direct and precise method for delivering stem cells into the infarcted region, with the highest rate of stem cell engraftment inside the determined region. However, the main disadvantage of the intramyocardial method is its highly invasive nature, which carries several safety concerns and can lead to complications such as cardiac perforation, arrhythmia induction, systemic embolization, and prolonged recovery time [36,37,40,43].

Cell delivery for cardiac treatment can be accomplished through various methods, such as the intramyocardial route, the retrograde coronary venous infusion route, and myocardial tissue engineering. The intramyocardial route is the most direct, but it comes with risks such as cardiac perforation and arrhythmia. Retrograde coronary venous infusion is done through coronary sinus injection, beneficial during cardiac surgery, but has limitations like coronary vein tortuosity. Myocardial tissue engineering, specifically with bioactive scaffolds, exhibits the potential to address obstacles such as inadequate cell viability [7-41]. Cell sheet transplantation, a novel application of cardiac tissue engineering, requires further investigation in animal models.

Mechanisms

The role of paracrine factors in cell-based cardiac repair is a topic of controversy. Paracrine factors are bioactive molecules that diffuse along short distances to induce changes in surrounding cells, contributing to cardiac repair through various mechanisms. These mechanisms include cardiomyocyte proliferation, promotion of cardiomyocyte cell cycle re-entry, cytoprotection and anti-apoptosis actions, differentiation of resident CSCs, angiogenesis, enhancing cardiac metabolism and contractility, anti-fibrosis actions, and immunomodulatory effects [51].

Injecting exogenous CSCs into the infarcted area of the myocardium may promote the proliferation of endogenous CSCs in both infarcted and noninfarcted regions. CSCs generate growth factors such as hepatocyte growth factor (HGF) and insulin growth factor-1 (IGF-1) that induce the migration of additional CSCs via the cardiac interstitium, proliferation, and differentiation into cardiomyocytes and vascular elements. This activation of endogenous CSCs has been proposed to have a role in the positive effects of other cell types, including MSCs [52].

Angiogenesis is another mechanism of benefit for stem cells, as proangiogenic factors such as vascular endothelial growth factor (VEGF), HGF, IGF-1, and angiopoietin are secreted by stem cells to promote neovascularization. Stem cells can also inhibit ventricular remodeling by altering extracellular matrix elements, restricting infarct expansion, LV remodeling, and myocardial fibrosis. Preventing ventricular hypertrophy is another mechanism of benefit, with stem cell cardiac therapy in HF models resulting in inhibition of the hypertrophic response of surviving cardiomyocytes. Paracrine factors like IGF-1 released by stem cells have been shown to enhance cardiomyocytes' survival and inhibit their apoptosis [51,52].

Cardiac regeneration involves the use of various genes, enzymes, and cytokines to enhance the functional recovery of the infarcted heart. C-Kit is a type III receptor tyrosine kinase involved in numerous intracellular signaling pathways, and it has been used as a marker to identify and enrich adult stem cells such as CSCs that are capable of differentiating into cardiomyocytes, endothelial cells, and smooth muscle cells in vitro and in vivo after myocardial infarction (MI). Pim-1 is another critical contributor to cardiac regeneration, expressed in stem cells, endothelial cells, and vascular smooth muscle cells. Genetic modification of CSCs and MSCs through increasing Pim-1 expression before injection into animal models has been associated with better cell engraftment and enhanced survival [53].

Combining growth factors with injected CSCs or MSCs is a novel approach to further enhance the functional recovery of the infarcted heart. Transforming growth factor (TGF-β) is a versatile and influential cytokine and growth factor that controls various cellular and metabolic reactions. It plays a role in reducing inflammation, modulating the immune system, promoting wound healing, stimulating the creation of new blood vessels, responding to cancer, and facilitating the regeneration of heart tissue. HIF-1 is a transcription factor responsible for the regulation of oxygen homeostasis, controlling cellular and systemic adaptive responses to hypoxia, and promoting angiogenesis, differentiation, survival, and protection of both transplanted and resident CSCs against the effects of hypoxia, inflammation, and reactive oxygen species in ischemic cardiac conditions [51-53].

Hepatocyte growth factor (HGF) and insulin-like growth factor-1 (IGF-1) are other examples of growth factors implemented in the process of cardiac regeneration. Combining HGF and IGF-1 with transplanted stem cells could be used as an adjuvant to cell injection for myocardial repair. The underlying mechanisms include promoting angiogenesis, differentiation, survival, and protection of both transplanted and resident CSCs against the effects of hypoxia, inflammation, and reactive oxygen species in ischemic cardiac conditions [54].

Heme oxygenase-1 (HO-1) is a Nrf2-regulated enzyme that protects against cellular injury, including heart damage. It catalyzes the breakdown of heme into CO, bilirubin, and ferrous ions. HO-1 increases VEGF, FGF, and IL-10 expression, while decreasing IL-1, IL-6, and TNF. It also generates angiogenic effects in MSCs and improves anti-oxidative and anti-apoptotic capacities [55].

Challenges Against Cell-Based Cardiac Repair and Possible Solutions

Challenges in cell-based cardiac repair include issues with stem cell collection, ex-vivo expansion, intracardiac delivery methods, cell dosage, and achieving adequate engraftment, differentiation, and functional improvement in vivo. Only a small percentage of transplanted stem cells survive and integrate into the host myocardium due to factors like ischemia, reperfusion injury, and inflammation. Strategies such as prosurvival stimulation before injection, embedding cells in bioactive scaffolds, and guided cardiopoiesis aim to improve engraftment and differentiation [46,52,53,56].

Clinical reproducibility poses a challenge, requiring preclinical studies in large animal models with physiological similarities to humans. Co-morbid conditions like hypertension, diabetes, and aging must be considered. Various clinical trials and meta-analyses have provided mixed results regarding the efficacy of stem cell therapy, with some showing benefits in terms of mortality reduction, improved cardiac function, and decreased hospital readmission, while others report no significant advantage [56].

Dosing and frequency of administration present uncertainties, as the relationship between cell dosage and therapeutic effects remains unclear. Studies suggest that dividing a given number of cells into multiple doses may be more effective than a single administration, emphasizing the importance of repeated dosing for maximum therapeutic efficacy. Further research is needed to establish the correlation between the number and frequency of cells delivered and their impact on heart function [57].

Possible Adverse Events Following Stem Cell Therapy

Stem cell therapy, while promising in terms of safety and efficacy, has reported adverse events in some studies. Short-term mortality in patients who received stem cell therapy was linked to various causes, such as mediastinitis, cardiogenic shock, intestinal ischemia, ventricular fibrillation, congestive heart failure (CHF), and myocardial ischemia. Long-term mortality was associated with events like cerebrovascular hemorrhage, sepsis after a heart transplant, heart failure, sudden cardiac death, and lung malignancy [58].

Arrhythmia, particularly with intramyocardial injection, is a frequently reported adverse effect. Potential mechanisms include lack of electromechanical integration, graft automaticity, direct injury and edema, transplantation of non-cardiomyocyte contaminants, nerve sprouting, immunologic reactions, and expression of gap junctions affecting arrhythmogenic potential. Paracrine factors were suggested to play a protective role against arrhythmia [59].

Occurrences of nonfatal myocardial infarctions were documented throughout both short-term and long-term monitoring after stem cell transplantation. Rehospitalization due to heart failure was also noted, with no significant difference compared to control groups in terms of the risk of readmission [60].

## Conclusions

Cell-based therapy, a promising treatment option for IHD and CHF, has the potential to revolutionize the treatment of these conditions. However, the exact role of stem cells in treating these conditions remains unexplored. Despite early trials showing potential improvements in heart function, concerns about the mechanism of action, cell dose, timing, and delivery methods remain unresolved. Large-scale clinical studies and approved technologies are crucial for the widespread adoption of stem cell treatment.
