# Correction: Stem Cell Therapy for Myocardial Infarction and Heart Failure: A Comprehensive Systematic Review and Critical Analysis

**DOI:** 10.7759/cureus.c259

**Published:** 2025-08-26

**Authors:** Mohamed R Abouzid, Ahmed Muaaz Umer, Suman Kumar Jha, Usman A Akbar, Own Khraisat, Amr Saleh, Kareem Mohamed, Sadaf Esteghamati, Ibrahim Kamel

**Affiliations:** 1 Internal Medicine, Baptist Hospitals of Southeast Texas, Beaumont, USA; 2 Internal Medicine Residency, Camden Clark Medical Center, Parkersburg, USA; 3 Internal Medicine, Sheer Memorial Adventist Hospital, Banepa, NPL; 4 Internal Medicine, Camden Clark Medical Center, Parkersburg, USA; 5 Internal Medicine, King Hussein Medical City, Amman, JOR; 6 Cardiovascular Medicine, Yale School of Medicine, New Haven, USA; 7 Internal Medicine, University of Missouri Kansas City, Kansas City, USA; 8 Internal Medicine, University of La Verne, La Verne, USA; 9 Internal Medicine, Steward Carney Hospital, Boston, USA

This article has been corrected to fix the following typo in the PRISMA diagram: "Studies included in review" has been corrected from 36 to 35.

